# Supplementary material for: Multi-Omics Integrative Analysis to Reveal the Impacts of Shewanella algae on the Development and Lifespan of Marine Nematode Litoditis marina
Source: Int J Mol Sci. 2024 Aug 22;25(16):9111. doi: 10.3390/ijms25169111 (PMC11354469; doi:10.3390/ijms25169111)
Supplement: Supplementary file 1 [file ijms-25-09111-s001.zip › Supplementary Information.pdf]

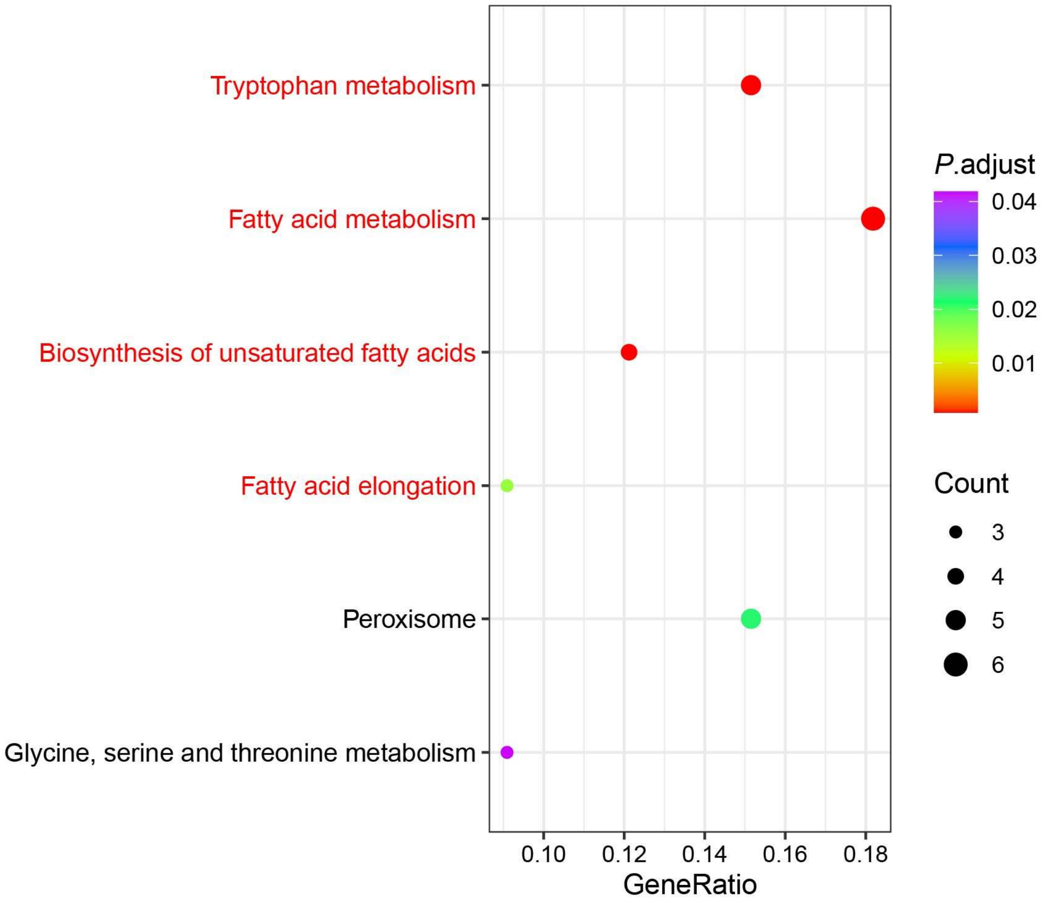


**Figure S1.** KEGG enrichment of upregulated DEGs in *L. marina* fed with *S. algae* versus *E. coli* OP50. The color from red to purple represents the significance of the enrichment. Details were shown in Table S5.

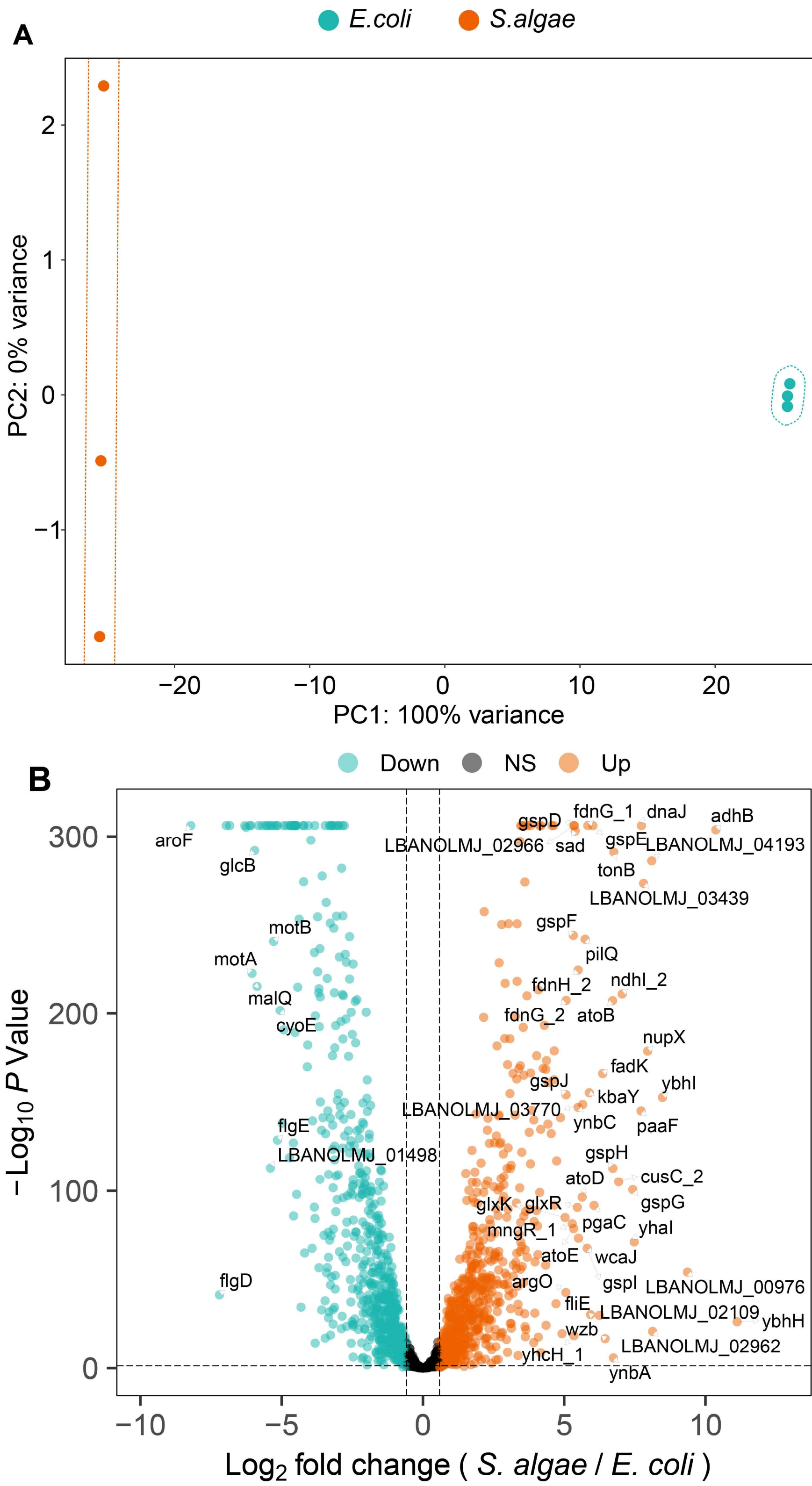


**Figure S2.** Transcriptomic characteristics of *S. algae* versus *E. coli* OP50. (A) Principal component analysis (PCA) of gene expression changes of *S. algae* versus *E. coli* OP50. (B) Volcano plots showing differentially regulated genes of

*S. algae* versus *E. coli* OP50. Up, upregulated genes; NS, genes with no significant changes; Down, downregulated genes.

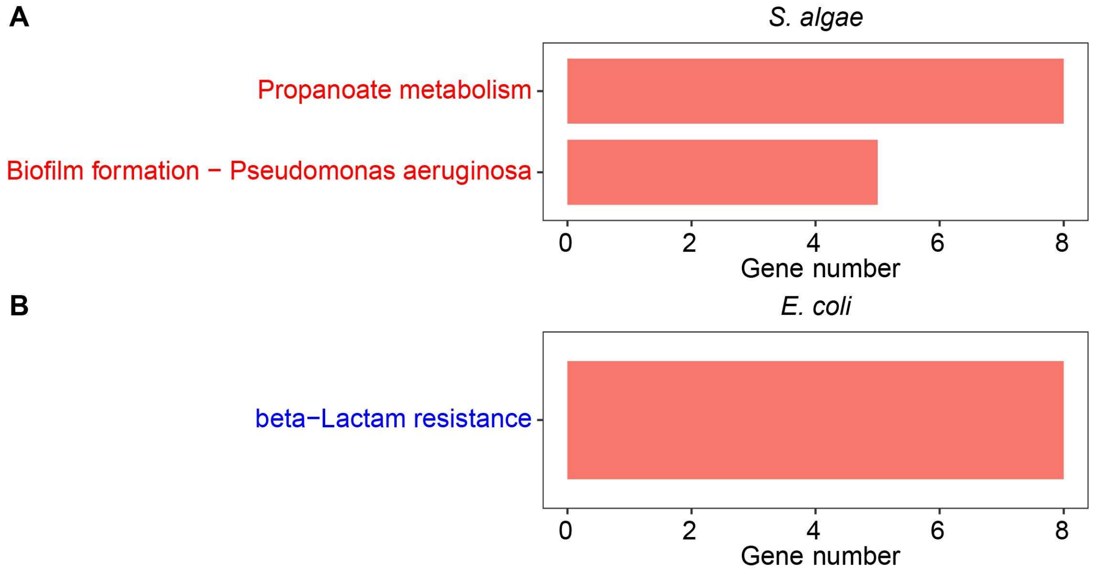


**Figure S3.** KEGG enrichment analysis for the top 10% expressed genes of *S. algae* and *E. coli* OP50.
